# THE ROLE OF SUBJECTIVE AGE IN PREDICTING POST-HOSPITALIZATION OUTCOMES

**DOI:** 10.1093/geroni/igac059.086

**Published:** 2022-12-20

**Authors:** Anna Zisberg, Nurit Gur-Yaish, Efrat Shadmi, Ksenya Shulyaev, Juliana Smichenko, Yuval Palgi

**Affiliations:** University of Haifa, Israel, Haifa, Hefa, Israel; Oranim Academic College of Education, Tivon, Hefa, Israel; University of Haifa, Haifa, Hefa, Israel; University of Haifa, Haifa, Hefa, Israel; University of Haifa, Haifa, Hefa, Israel; University of Haifa, Haifa, Hefa, Israel

## Abstract

Subjective age contributes to a range of health and functional outcomes in older adults. Most of the evidence comes from studies in community dwelling older adults. The current study explores whether younger subjective age serves as a protective factor against hospital associated physical, cognitive, and emotional decline. This paper is a secondary analysis of a subsample (N=250) from the HoPE-MOR (Hospitalization Process Effects on Mobility Outcomes and Recovery) study for which subjective age was assessed at the time of hospital admission and outcomes were measured one-month post-discharge. Psychological and physiological subjective age was measured as a person’s report on the degree to which they feel older or younger compared to their chronological age on a 5-point Likert-type scale. Measures of independency in Activities of Daily Living, Life-space mobility, cognitive function and depressive symptoms, were based on participants’ assessment at admission and one-month post-discharge. In a sample of acutely ill participants, age 77.5±6.6, those with younger psychological subjective age had a significantly lower odds for poorer mental (OR=0.66, 95%CI 0.45-0.97), functional (OR=0.62, 95%CI 0.43-0.90) and cognitive state (OR=0.60, 95%CI 0.36-0.98), and better life-space mobility (OR=0.67, 95%CI 0.47-0.95). Findings were significant after controlling for numerous demographic, functional, cognitive, emotional and chronic and acute health predictors. Physiological subjective age was not significantly related to post hospitalization outcomes. Psychological subjective age could serve as a relatively simple parameter to identify older adults who are at risk for poor hospitalization outcomes for consideration of inclusion in preventive in-hospital and post discharge interventions.

